# Bacterial Spinal Epidural and Psoas Abscess in Pregnancy Associated with Intravenous Drug Use

**DOI:** 10.1155/2018/1797421

**Published:** 2018-06-11

**Authors:** Tirtza N. Spiegel Strauss, Sarah L. Pachtman, Burton Rochelson

**Affiliations:** Division of Maternal Fetal Medicine, Department of Obstetrics and Gynecology, The Donald and Barbara Zucker School of Medicine at Hofstra/Northwell, North Shore University Hospital, Manhasset, NY, USA

## Abstract

Spontaneous spinal epidural abscess (SEA) is a rare infection of the central nervous system. We report a case of a 25-year-old G3 P0020 at 36 weeks of gestational age with history of intravenous drug abuse presenting with acute-onset and severe back pain. Despite antibiotic therapy, pain worsened and she developed lower extremity weakness. Magnetic resonance imaging (MRI) revealed an SEA, and cesarean delivery was performed secondary to increasing weakness, followed by laminectomy (T9-12) and decompression of epidural abscess. Postoperative course was complicated by a psoas muscle abscess and persistent SEA refractory to antibiotic therapy, requiring surgical reexploration and extended treatment with antibiotics. She was discharged home in stable condition and neonate did well with no resulting sequelae. Spinal epidural and psoas abscesses are rare and diagnosis is often delayed. Prompt recognition and treatment are necessary to prevent catastrophic neurologic consequences, and the diagnosis should be considered in pregnant patients presenting with back pain, especially in those with risk factors.

## 1. Introduction

Back pain is a common complaint in pregnancy; when it is severe and persistent despite analgesia or is accompanied by neurologic symptoms, factors unrelated to pregnancy should be considered. Spontaneous spinal epidural abscess (SEA) is a rare infection of the central nervous system that can cause lower back pain, with few cases in pregnancy reported in the literature. In the obstetric population, it is most frequently associated after regional anesthesia is administered for delivery, and the diagnosis is often not considered in pregnancy. Other possible risk factors for development of SEA include immunodeficiency, diabetes mellitus, bacteremia, trauma, or intravenous drug use [1, 2]. We report the case of a woman who developed an epidural abscess in the third trimester of pregnancy requiring urgent cesarean delivery due to worsening neurologic status, followed by laminectomy for surgical management of epidural abscess.

## 2. Case

A 25-year-old G3 P0020 at 36 3/7 weeks of gestational age with a singleton pregnancy presents with acute-onset, severe back pain and fever. Pain was described as constant, aching, and sharp. Her prenatal course was significant for multiple left antecubital abscesses requiring drainage (culture positive for methicillin-resistant staphylococcus aureus, MRSA) at 34 weeks and she was treated with clindamycin. On initial questioning, she admitted daily tobacco use but denied intravenous drug use. She was afebrile on presentation, but nodularity was appreciated at the left antecubital fossa and she had lower back tenderness to palpation. Physical exam was otherwise unremarkable. Biophysical profile and nonstress test confirmed a reassuring fetal status.

Initial white blood cell count (WBC) was 21 [K/uL], C-reactive protein (CRP) was 27 [mg/L], and erythrocyte sedimentation rate (ESR) was 63 [mm/hour]. Urine toxicology screening was negative. Empiric treatment with vancomycin and piperacillin/tazobactam was initiated after she developed hypotension and a fever. Preliminary blood cultures were positive for gram positive cocci, later found to be positive for MRSA. Magnetic resonance imaging (MRI) of lumbar spine was obtained because of severe lower back pain that did not resolve with analgesia. This study revealed a small dorsal spinal collection with edema in the left psoas muscle. Neurologic reflexes were intact and serial neurologic exams were normal.

**Figure 1:**
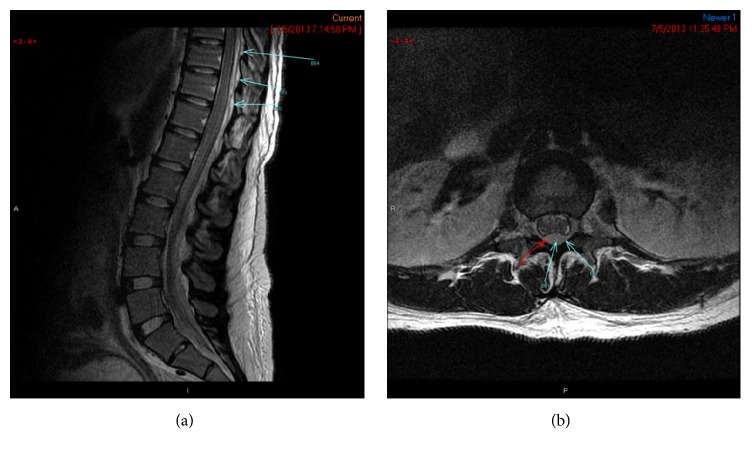
Images of SEA pretreatment.

Back pain continued to increase, and the patient developed weakness of bilateral lower extremities. Given the concern for acute structural damage to the spinal cord, the patient was counseled regarding risks, benefits, and alternatives to contrast imaging during pregnancy and opted for MRI with gadolinium intravenous contrast [3]. This was repeated after two days of antibiotic therapy to assess for further progression of abscess. MRI revealed an epidural abscess from T5-6 to T8-9 causing mild thecal cord compression and a collection from L4 to S1 (see Figures 1(a) and 1(b)). Because of concern for worsening neurologic symptoms of lower extremity weakness and difficulty ambulating, cesarean delivery was performed followed by laminectomy (T5-10) and decompression of the epidural abscess. Intraoperative cultures were obtained and were positive for MRSA. Cesarean delivery was uncomplicated. Neonate weighed 2690 grams with Apgar score of 8 and 9 and was treated with vancomycin and gentamycin for suspected sepsis. Placental pathology and cultures were negative.

**Figure 2:**
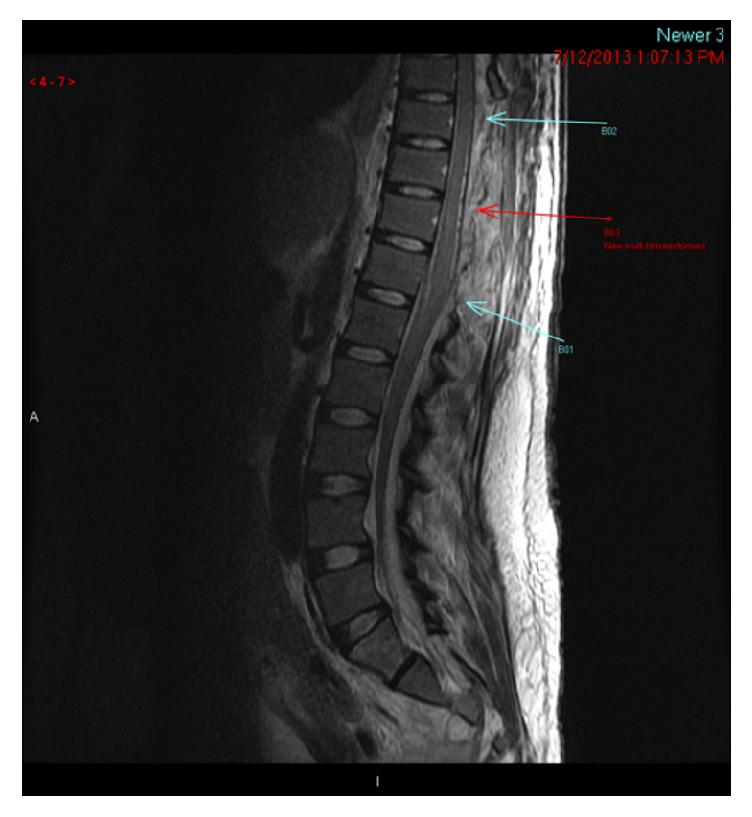
Image of SEA posttreatment.

Postoperative course was complicated by persistent MRSA bacteremia. Repeat MRI revealed residual abscess from T6-T9, with cord compression at T6-T7, and a left psoas muscle collection (see Figure 2). Three days later, reexploration and laminectomy from T4-T7 were performed and antibiotic treatment was continued with vancomycin and ceftaroline fosamil. During her extended hospital course, she admitted to previous illicit drug use but denied current use. However, there was a high suspicion for intravenous drug use due to her poor pain tolerance and history of antecubital abscesses. Later in her clinical course she endorsed a history of intravenous drug use. Once blood cultures were negative, she was discharged home in stable condition to continue long-term antibiotic therapy. Lower extremity weakness had resolved at time of discharge; however, she continues to have residual weakness, pain, and incontinence.

## 3. Discussion

Back pain is common in pregnancy, but severe and persistent back pain, along with other symptoms, should prompt a thorough investigation for other causes. Using MRI to expedite diagnosis can prevent lingering neurologic sequelae that are common complications of SEA. This case was also complicated by psoas muscle abscess that led to difficulty in treatment of this complicated infection. Prompt recognition and management of SEA abscess in pregnancy, along with appropriate management for gestational age, may lead to optimal maternal and fetal outcomes. Risk factor screening and targeted history taking can assist in timely diagnosis and are essential for clues to construct a differential diagnosis of common presentations in pregnancy.

SEAs are most common in thoracolumbar areas, where the epidural space is larger and contains fatty tissue [4]. Bacteria may enter the epidural space via a hematogenous route or local extension from surrounding infection. Most SEAs contain pus or granulation tissue and occur posteriorly and tend to extend vertically (average is 3-5 spinal cord segments) causing direct compression or thrombosis, which may lead to severe neurologic deficits [5].

Staphylococcus aureus is the leading pathogen isolated in SEA [1]. Risk factors include diabetes, human immunodeficiency virus (HIV) infection, trauma, bacteremia, intravenous drug abuse (IVDA), and contiguous bony or soft tissue infection [6]. In this case, the patient was thought to have no known risk factors because she denied IVDA, and epidural abscess was diagnosed after MRI. Targeted screening is critically important in pregnant patients to facilitate consideration of risk factors that are becoming more common in pregnant patients. Intravenous drug use is increasing in pregnant patient populations, and high suspicion for this risk factor is important [7, 8].

Symptoms of SEA include fever, back pain, and neurologic complaints such as vision changes, paresthesias, and weakness. Patients rarely present with all three symptoms, which delays diagnosis and can ultimately cause progression to paralysis. Thus, if spinal epidural abscess is suspected or risk factors are present during the antepartum period, urgent imaging studies are warranted to expedite diagnosis. MRI has been shown to provide optimal visualization [6]. There are no consistent laboratory findings that are predictive of SEA. In one study, ESR was elevated in 100% of patients with spine pain and another risk factor for SEA (and in only 33% of patients without SEA) [9]. Broad-spectrum antibiotics are primary therapy. However, surgical intervention may be required to prevent severe neurologic sequelae caused from spinal compression [1, 10]. The entire spine should be imaged as skip lesions may occur.

Spontaneous SEA has been reported in few cases of pregnant patients [11–17]. All cases have occurred in women between 20 and 30 years of age (compared to median age of 50 in the general population), ranging from 20 to 34 weeks of gestational age, who have presented with neurological symptoms (with or without fevers). All but one of these patients required laminectomies, and the majority of patients had complete resolution of neurologic symptoms.

Spinal epidural abscesses are very rare. However, the incidence has increased over time, possibly due to increasing diagnostic accuracy with MRI [18]. As the opioid epidemic continues to increase and intravenous drug abuse becomes more prevalent in the pregnant population, SEA should be considered more readily when women present with fever, back pain, or neurologic complaints, and the appropriate workup should be performed.
